# A Case of Pathological Complete Response Following FOLFIRINOX Therapy for Pancreatic Adenocarcinoma with Synchronous Distant Lymph Node Metastases

**DOI:** 10.1016/j.ijscr.2020.06.044

**Published:** 2020-06-13

**Authors:** Masanori Tsujie, Soichi Fumita, Tomoko Wakasa, Shigeto Mizuno, Hajime Ishikawa, Kotaro Kitani, Shumpei Satoi, Kaoru Okada, Keisuke Inoue, Shuichi Fukuda, Hironobu Manabe, Noriko Ichimura, Shinya Ueda, Takao Tamura, Toshihiko Kawasaki, Masao Yukawa, Yoshio Ohta, Masatoshi Inoue

**Affiliations:** a Department of Gastroenterological Surgery, Kindai University Nara Hospital, Japan; b Department of Medical Oncology, Kindai University Nara Hospital, Japan; c Department of Pathology, Kindai University Nara Hospital, Japan; d Department of Endoscopic Diagnosis and Treatment, Kindai University Nara Hospital, Japan; e Department of Nursing, Kindai University Nara Hospital, Japan; f Department of Gastroenterology, Kindai University Nara Hospital, Japan; g Department of Surgery, Osaka Rosai Hospital, Japan

**Keywords:** CA19-9, carbohydrate antigen 19-9, CEA, carcinoembryonic antigen, CT, computed tomography, FOLFIRI, 5-fluorouracil, leucovorin, and irinotecan, FOLFIRINOX, 5-fluorouracil, leucovorin, irinotecan, and oxaliplatin, pCR, pathological complete response, PDAC, panceatic ductal adenocarcinoma, PET, positron emission tomography, Case report, Unresectable pancreatic cancer, Distant metastasis, FOLFIRINOX, Conversion surgery, BRCA1/2 mutation

## Abstract

**Introduction:**

We report a case of conversion surgery for pancreatic ductal adenocarcinoma (PDAC) with synchronous distant metastases showing pathological complete response (pCR) after FOLFIRINOX therapy.

**Presentation of case:**

A 46-year-old woman with obstructive jaundice was referred to our hospital. A CT scan revealed a hypo-vascular mass in the head of the pancreas with multiple para-aortic lymph nodes and a Virchow’s node swollen. The serum CA 19-9 level was 71795.1 U/mL. The result of tumor biopsy from the biliary stenotic site was concordant with adenocarcinoma. She was diagnosed with PDAC with distant metastases. After 10 courses of FOLFIRINOX followed by 4 courses of FOLFIRI, a CT scan showed that distant lymph node swellings disappeared, and CA19-9 level became almost normal. She underwent pancreaticoduodenectomy with dissection of para-aortic lymph nodes 8 months after the initiation of chemotherapy. Pathologically, no evidence of residual adenocarcinoma was observed in neither pancreas nor lymph nodes. Adjuvant chemotherapy using S-1 was administered for 6 months, and no recurrence has been observed 4 years after surgery. BRCA1/2 mutations were not detected in patient’s DNA.

**Discussion:**

With the induction of intensive chemotherapies such as FOLFIRINOX, an increasing number of patients with synchronous distant metastases could become suitable candidates for surgery of the primary lesion because of the potential complete response of metastatic lesions.

**Conclusion:**

This case presented a rare occurrence of pCR in a patient with unresectable PDAC with distant metastases who received FOLFIRINOX. The feasibility and benefits of conversion surgery in such patients must be investigated in future trials.

## Highlights

• The patient was diagnosed with PDAC with distant lymph node metastases (para-aortic lymph nodes and Virchow’s lymph node).
• After systemic chemotherapy using FOLFIRINOX, conversion surgery (pancreaticoduodenectomy) was performed.
• Pathologically, no evidence of residual adenocarcinoma was observed in neither pancreas head nor dissected lymph nodes.
• No recurrence has been observed 4 years after conversion surgery.
• BRCA1/2 mutations were not detected in patient’s DNA extracted from blood.

## 1 Introduction

Pancreatic ductal adenocarcinoma (PDAC), which constitutes 90% of pancreatic cancers, is the fourth leading cause of cancer-associated mortality worldwide [1,2]. Although surgical resection is the only potential curative treatment currently available, 35% of the patients have unresectable locally advanced disease and 50% have distant metastatic disease at the time of diagnosis, and curative resection cannot have been chosen for those patients [3]. Especially in those with metastases from PDAC, the median survival remains less than 1 year and survival beyond even 2 years is rare. Recently, with the induction of intensive chemotherapy regimens such as FOLFIRINOX (combination of 5-fluorouracil, leucovorin, irinotecan, and oxaliplatin) and gemcitabine plus nab-paclitaxel for the treatment of metastatic pancreatic cancer patients, antitumor activity and overall survival in those patients have dramatically improved [4,5]. These advances in chemotherapy have led to the possibility of conversion of unresectable disease to resectable disease, and it has been reported that more than 20% of pancreatic cancer patients with locally advanced disease at diagnosis undergo successful conversion surgery after FOLFIRINOX treatment [6,7]. However, even in those cases, pathological complete response (pCR) following FOLFIRINOX therapy is extremely rare. Moreover, there are few reports of conversion surgery for unresectable PDAC with distant metastases. In this case report, we present the case of a patient with synchronous distant lymph node metastases from PDAC who achieved pCR after FOLFIRINOX followed by FOLFIRI (combination of 5-fluorouracil, leucovorin, and irinotecan), showing 4-year recurrence-free survival after conversion surgery. This work has been reported in line with the SCARE criteria [8].

## 2 Presentation of case

**Fig. 1:**
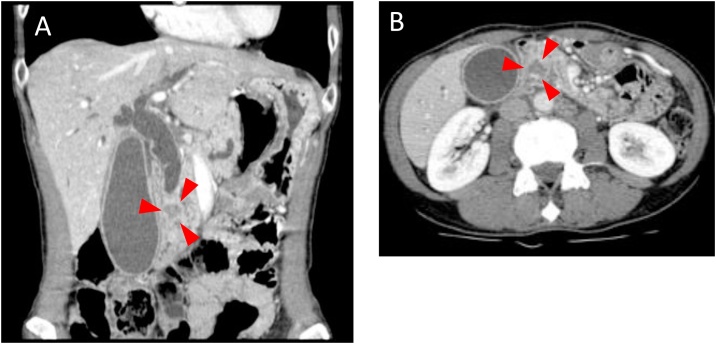
Computed tomography (CT) scan showing pancreatic head tumor (red arrow heads) causing dilatation of biliary and pancreatic ducts. A: axial view; B: coronal view.

**Fig. 2:**
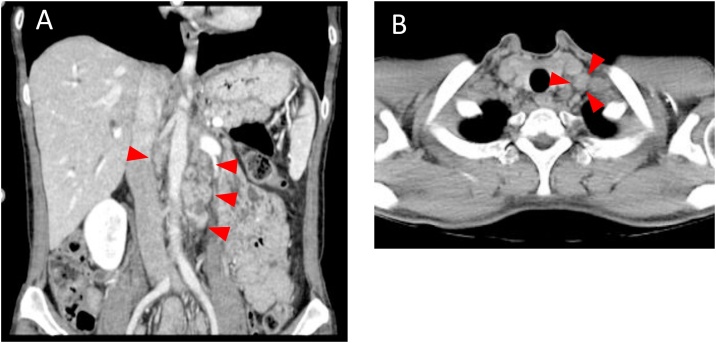
Computed tomography (CT) scan showing swellings of distant lymph nodes (red arrow heads). A: para-aortic lymph nodes (red arrow heads); B: left supraclavicular node (red arrow heads).

**Fig. 3:**
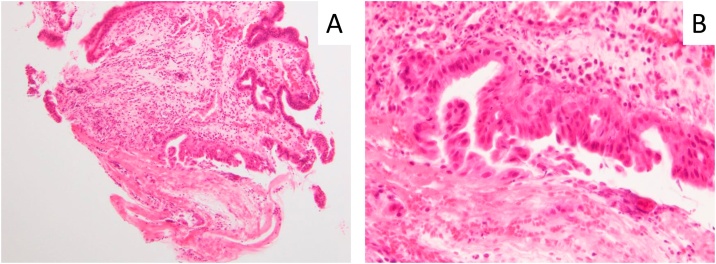
Histopathological findings of biopsy specimen consistent with well differentiated adenocarcinoma (hematoxylin-eosin staining). A: low magnification; B: high magnification.

**Fig. 4:**
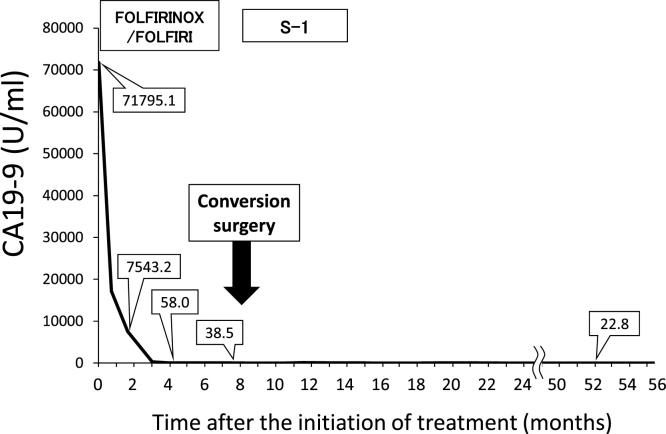
Transition graph of serum carbohydrate antigen 19-9 (CA19-9) level and the treatment progress.

**Fig. 5:**
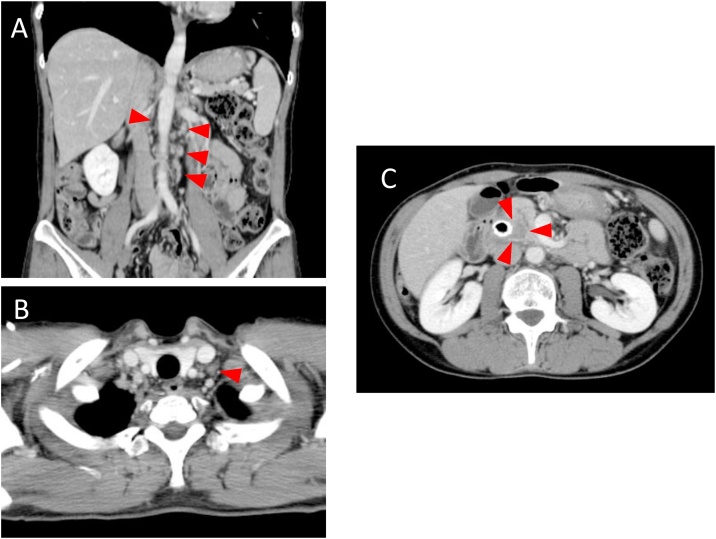
Computed tomography (CT) scan after FOLFIRINOX (combination of 5-fluorouracil, leucovorin, irinotecan, and oxaliplatin) treatment showing only tiny appearances of distant lymph nodes. A: para-aortic lymph nodes (red arrow heads); B: left supraclavicular lymph node (a red arrow head); C: primary lesion (red arrow heads).

**Fig. 6:**
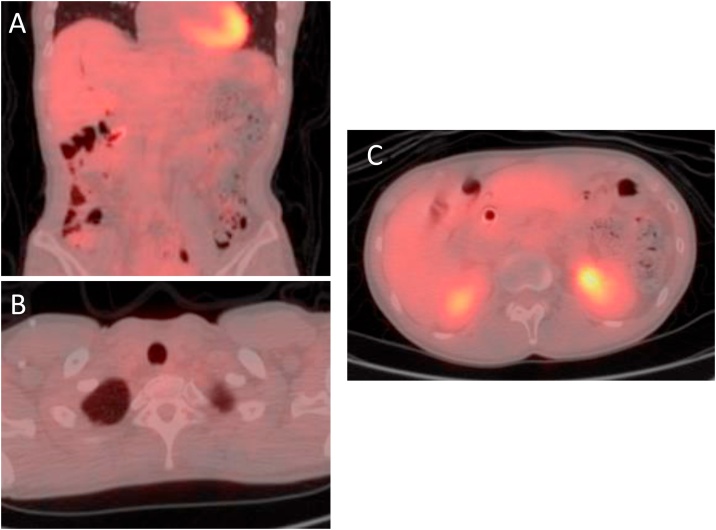
Positron emission tomography (PET)-CT scan just before conversion surgery showing no distant metastases. A: para-aortic lymph nodes; B: left supraclavicular lymph node; C: primary lesion.

A 46-year-old female patient with no past medical history visited a previous hospital due to obstructive jaundice. An abdominal computed tomography (CT) scan revealed a hypo-vascular mass with 16-mm in diameter located in the head of the pancreas causing dilatation of biliary and pancreatic ducts (Fig. 1A, B). The swelling of multiple para-aortic lymph nodes and a left supraclavicular lymph node (Virchow’s node) was also observed (Fig. 2A, B). She was suspected of having pancreatic cancer with distant lymph node metastases. After undergoing endoscopic retrograde biliary drainage with a plastic stent, she was referred to our hospital for further examination and treatment. The serum level of carbohydrate antigen 19-9 (CA19-9), carcinoembryonic antigen (CEA), and DUPAN-2 was 71795.1 U/mL (normal range: <37 U/mL), 47.9 ng/mL (normal range: <5 ng/mL), and 8611 U/mL (normal range: <150 U/mL), respectively. For histopathological diagnosis, she underwent endoscopic retrograde cholangio-pancreatography. Because the cannulation of main pancreatic duct was incapable, transluminal biopsy from the biliary stenotic site was performed after removing the inserted plastic stent. A metallic stent for biliary drainage was inserted at the same time. The result of biopsy was concordant with well differentiated tubular adenocarcinoma (Fig. 3A, B). According to these findings, she was diagnosed with unresectable pancreatic adenocarcinoma with distant lymph node metastases. After placement of a central venous port, FOLFIRINOX (oxaliplatin 85 mg/m^2^, leucovorin 200 mg/m^2^, irinotecan 180 mg/m^2^, 5-fluorouracil 400 mg/m^2^ given as a bolus followed by 2400 mg/m^2^ given as a 46-h continuous infusion, all on day 1, and then repeated every 2 weeks) was initiated as first-line chemotherapy. After 4 administrations of FOLFIRINOX (2 months after the initiation of chemotherapy), a CT scan showed marked tumor reduction of distant metastases although it was very difficult to evaluate the response of the primary lesion because of the metallic stent. Serum CA19-9 level was sharply decreased to 7543.2 U/mL (almost 90% reduction) (Fig. 4). After 6 more administrations of FOLFIRINOX (10 cycles in total), a CT scan revealed that swellings of distant lymph nodes almost disappeared, and serum level of CA19-9 decreased to 58.0 U/mL (Figs. 4, 5A, B). Because of worsening neurotoxicity caused by oxaliplatin, the patient received another 4 courses of FOLFIRI (irinotecan 180 mg/m^2^, leucovorin 200 mg/m^2^, 5-fluorouracil 400 mg/m^2^ given as a bolus followed by 2400 mg/m^2^ given as a 46-h continuous infusion, all on day 1, and then repeated every 2 weeks). A CT scan revealed that shrunken lymph nodes had remained stable, and serum CA19-9 level became almost normal (38.5 U/mL) (Fig. 4). A positron emission tomography (PET)-CT scan also showed no abnormal uptake in distant lymph nodes (Fig. 6A, B). As to primary lesion, significant uptake was not observed in PET-CT although hypo-vascular mass remained left side of the metallic stent (Fig. 5C, 6C).

**Fig. 7:**
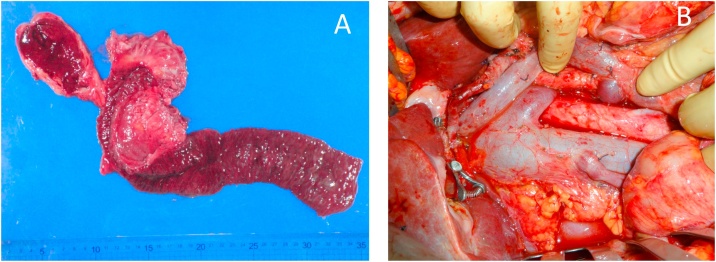
Surgical specimen of subtotal stomach preserving pancreaticoduodenectomy (A) and an intraoperative picture after dissection of para-aortic lymph nodes (B).

**Fig. 8:**
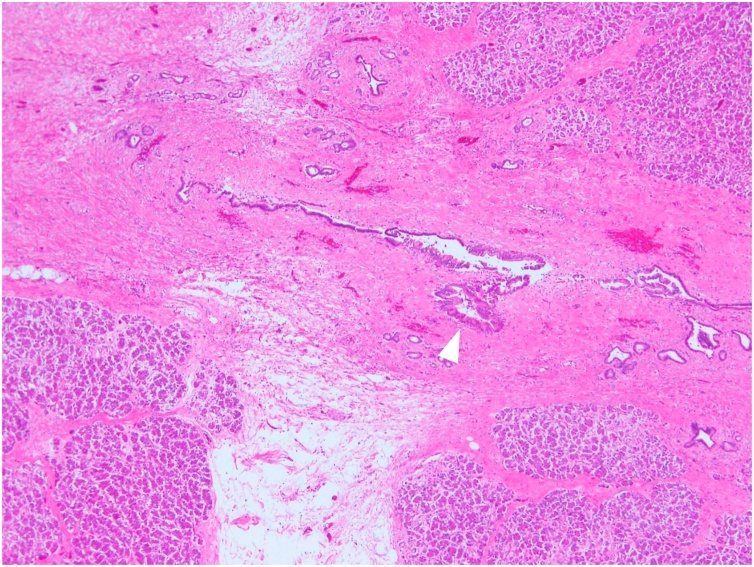
Histopathological findings of surgical specimen showing scattered foci of grade IB pancreatic intraepithelial neoplasia (hematoxylin-eosin staining, x100) (a white arrow).

Given these results, and after discussion at multidisciplinary cancer conference, the patient underwent subtotal stomach preserving pancreaticoduodenectomy with dissection of para-aortic lymph nodes 8 months after the initiation of chemotherapy (Fig. 7A, B). Histopathological examination of the surgical specimen showed no evidence of residual adenocarcinoma in neither pancreas head nor 54 dissected lymph nodes including para-aortic nodes, although scattered foci of grade IB pancreatic intraepithelial neoplasia were observed (Fig. 8). These findings were consistent with pathological complete response (pCR) to the treatment. The effect of chemotherapy was Grade III in Evans classification [9]. The postoperative course was uneventful, and she was discharged on day 27 after surgery. Adjuvant chemotherapy using S-1 was administered for 6 months in outpatient clinic, and no recurrent sign has been observed 4 years after conversion surgery. Because platinum-based chemotherapy regimens have been reported to be associated with superior overall survival in PDAC patients with germline BRCA1/2 mutations, we performed the germline test after she received genetic counseling. However, germline BRCA1/2 mutations were not detected in patient DNA extracted from a blood sample.

## 3 Discussion

Pancreatic ductal adenocarcinoma (PDAC) remains a very aggressive disease with an overall 5-year survival of only 5% [2]. Para-aortic lymph nodes involvement is generally considered as distant metastases and surgical resection should not be recommended [10]. In our case, as the tumor metastasized to not only para-aortic lymph nodes but also a left supraclavicular lymph node, systemic chemotherapy was chosen as a first-line treatment instead of radical surgery.

Recently, FOLFIRINOX therapy has become one of the first-line chemotherapeutic regimens for metastatic pancreatic cancer [4]. Some reports the experience of conversion surgery of unresectable pancreatic cancer with metastasis after FOLFIRINOX treatment. Furuse et al. experienced the surgical resection of initially unresectable pancreatic cancer with liver metastases which showed complete response after FOLFIRINOX treatment [7]. Schneither et al. reported that two pancreatic cancer patients with liver metastasis underwent conversion surgery after FOLFIRINOX treatment [11]. In the first case, no cancer cells were detected in the resected liver, and no recurrence has been observed in the liver for 16 months after conversion surgery. In the second case, the patient has not shown recurrent hepatic metastases for 9 months after surgery although hepatectomy was not performed. Our case also showed no recurrence at the left supraclavicular lymph node 4 years after conversion surgery although we did not perform Virchow’s node dissection. In pancreatic cancer, majority of tumor volume is made of stroma, and the desmoplastic stroma serves as a physical barrier of drug delivery into the tumor, causing refractoriness to chemotherapy [12,13]. Therefore, stroma-poor metastatic lesions could show better response to chemotherapy than stroma-rich primary lesions. With the induction of intensive chemotherapies such as FOLFIRINOX, an increasing number of such patients with synchronous distant metastases could become suitable candidates for surgery of the primary lesion because the potential complete response of metastatic lesions.

It remains unclear that appropriate patient selection and optimal timing of conversion surgery in pancreatic cancer patients with distant metastasis who showed favorable response to non-surgical treatment. Satoi et al. reported the safety and efficacy of conversion surgery in initially unresectable pancreatic cancer patients with a favorable response to chemotherapy and/or chemoradiotherapy over 6 months after the initial treatment [14]. Out of 58 patients treated with conversion surgery, 17 patients had distant metastasis (13 liver metastasis, 3 distant lymph node metastasis, and 1 peritoneal dissemination), and their median survival time after initial treatment was 39 months, which was much longer than 19 months in control group. They also showed a significantly longer overall survival in patients who underwent conversion surgery over 240 days after the initial treatment than those less than 240 days. Wright et al. investigated the outcomes of PDAC patients with distant metastases who underwent primary tumor resection with or without metastatectomy following a favorable response to systemic chemotherapy including FOLFIRINOX and gemcitabine-based regimens [15]. Median time from diagnosis to surgery and median overall survival were 9.7 and 34.1 months, respectively, in 23 patients who met the study criteria. Although those patients in two reports were retrospectively selected super-responders to the initial treatment, conversion surgery could have an important role in multidisciplinary treatment for patients with distant metastasis. Our patient showed a favorable response to the FOLFIRINOX/FOLFIRI treatment over 6 months and underwent conversion surgery 8 months after initial treatment. No recurrence has been observed 4 years after surgery, which could support their data thus far.

Several reports have shown that platinum-based chemotherapy treatment regimens are associated with superior overall survival in PDAC patients with germline BRCA1/2 mutations [[16], [17], [18]]. Therefore, we conducted the germline examination after genetic counseling. However, germline mutations of BRCA1/2 were not detected in her DNA. Her good response to FOLFIRINOX has resulted in no association with BRCA1/2 mutations.

The prognostic value of reduction rate of CA19-9 level during chemotherapy has been reported in a number of papers. They demonstrated that a decrease of CA19-9 > 20–50% during 8 weeks of chemotherapy is associated with a better survival of patients with locally advanced or metastatic pancreatic cancer [14,[19], [20], [21], [22]]. In our case, the serum CA19-9 concentration decreased by 89.4% (from 71795.1 U/mL to 7543.2 U/mL) two months after treatment initiation, and the serum CA19-9 level has been within normal range for more than 3 years after conversion surgery. This dramatic decrease of CA19-9 could have reflected the long disease-free survival of our patient.

Left sided supraclavicular nodes, also termed as Virchow’s nodes, represent a well-characterized site of metastasis, especially in the setting of gastrointestinal malignancies, but a rare site of metastasis in pancreatic cancer. A review of literature reveals only 11 cases of supraclavicular metastasis from pancreatic adenocarcinoma [[23], [24], [25]]. Interestingly, four cases out of 11 had isolated supraclavicular lymph node metastasis without spread to usual sites of metastasis in pancreatic cancer such as the liver and peritoneal cavity, which is same as our experience.

## 4 Conclusion

The current work presented a rare occurrence of pCR in a patient with unresectable pancreatic cancer with distant metastases following treatment of FOLFIRINOX therapy. The feasibility and benefits of conversion surgery in such patients must be investigated in future trials.

## Declaration of competing interest

The authors declare that they have no conflicts of interest.

## Sources of funding

Not applicable.

## Ethical approval

The institutional Ethics Committees of Kindai University Nara Hospital approved publication of this case report.

## Consent

Written informed consent was obtained from the patient for publication of this case report.

## Author contribution

MT participated in the care of the patient including the operation and wrote the initial draft of the manuscript. SF, SU, and TT participated in the chemotherapy and revised the manuscript. TW and YO prepared the pathological findings and revised the manuscript. SM participated in the endoscopic examination and revised the manuscript. HI and MY participated in the surgery and revised the manuscript. NI assisted the patient in receiving genetic counseling and having genetic testing. KK, SS, KO, KI, SF, HM, TK, and MI participated in the discussion and critically reviewed the manuscript. All authors read and approved the final manuscript.

## Registration of research studies

Not applicable.

## Guarantor

Masanori Tsujie M.D., Ph.D.

## Provenance and peer review

Not commissioned, externally peer-reviewed.
